# Quality of life measures predict cardiovascular health and physical performance in chronic renal failure patients

**DOI:** 10.1371/journal.pone.0183926

**Published:** 2017-09-14

**Authors:** A. Rogan, K. McCarthy, G. McGregor, T. Hamborg, G. Evans, S. Hewins, N. Aldridge, S. Fletcher, N. Krishnan, R. Higgins, D. Zehnder, S. M. Ting

**Affiliations:** 1 Department of Emergency Medicine, Wellington Hospital, Capital and Coast District Health Board, Wellington, New Zealand; 2 Department of Renal Medicine and Transplantation, University Hospital Coventry and Warwickshire NHS Trust, Coventry, United Kingdom; 3 Department of Cardiac Rehabilitation and Cardiology, University Hospital Coventry and Warwickshire NHS Trust, Coventry, United Kingdom; 4 Division of Health Sciences Statistics and Epidemiology, University of Warwick, Coventry, United Kingdom; 5 Department of Acute Medicine, North Cumbria University Hospital NHS Trust, Carlisle, United Kingdom; 6 Division of Translational Medicine, University of Warwick, Coventry, United Kingdom; 7 Department of Acute Medicine, Heart of England NHS Foundation Trust, Birmingham, United Kingdom; Hospital Universitario de la Princesa, SPAIN

## Abstract

**Background:**

Patients with advanced chronic kidney disease (CKD) experience complex functional and structural changes of the cardiopulmonary and musculoskeletal system. This results in reduced exercise tolerance, quality of life and ultimately premature death. We investigated the relationship between subjective measures of health related quality of life and objective, standardised functional measures for cardiovascular and pulmonary health.

**Methods:**

Between April 2010 and January 2013, 143 CKD stage-5 or CKD5d patients (age 46.0±1.1y, 62.2% male), were recruited prospectively. A control group of 83 healthy individuals treated for essential hypertension (HTN; age 53.2±0.9y, 48.22% male) were recruited at random. All patients completed the SF-36 health survey questionnaire, echocardiography, vascular tonometry and cardiopulmonary exercise testing.

**Results:**

Patients with CKD had significantly lower SF-36 scores than the HTN group; for physical component score (PCS; 45.0 vs 53.9, p<0.001) and mental component score (MCS; 46.9 vs. 54.9, p<0.001). CKD subjects had significantly poorer exercise tolerance and cardiorespiratory performance compared with HTN (maximal oxygen uptake; VO_2_peak 19.9 vs 25.0ml/kg/min, p<0.001). VO_2_peak was a significant independent predictor of PCS in both groups (CKD: *b* = 0.35, p = 0.02 vs HTN: *b* = 0.27, p = 0.001). No associations were noted between PCS scores and echocardiographic characteristics, vascular elasticity and cardiac biomarkers in either group. No associations were noted between MCS and any variable.

The interaction effect of study group with VO_2_peak on PCS was not significant (ΔB = 0.08; 95%CI -0.28–0.45, p = 0.7). However, overall for a given VO_2_peak, the measured PCS was much lower for patients with CKD than for HTN cohort, a likely consequence of systemic uremia effects.

**Conclusion:**

In CKD and HTN, objective physical performance has a significant effect on quality of life; particularly self-reported physical health and functioning. Therefore, these quality of life measures are indeed a good reflection of physical health correlating highly with objective physical performance measures.

## Introduction

The sense of well-being and ability to function productively in daily life is poor in patients with advanced chronic kidney disease (CKD) compared to the general population [1–4]. For patients with CKD, impaired quality of life is a consistent predictor of more objective patient outcomes [5–10].

Health related quality of life (HRQoL) assessment of patients is becoming an increasingly important clinical tool. One of the most widely used generic HRQoL instruments in CKD is the Medical Outcomes Study 36-Item Instrument Short Form Health Survey (SF-36)[5, 8–11]. The SF-36 assesses eight generic scales of HRQoL. Two composite measures, the physical component summary (PCS) and the mental component summary (MCS), are derived from the eight scales [12].

Patients with advanced CKD are at high risk of cardiovascular disease (CVD). Complex changes in both the cardiac and vascular systems results in structural and functional changes that can lead to reduced exercise tolerance, quality of life and ultimately premature death [13–16].

Exercise capacity relies on the health of the circulatory system. The association between the somewhat subjective measures of a self-reported HRQoL and more objective measures such as cardiac structural and functional measures are not well understood. A reduction in exercise capacity limits the range of physical activities that a patient can accomplish. The importance of this is underscored by a previously reported association between mortality and physical activity assessed through questionnaires in CKD patients[3, 4, 8, 9]. A similar association was also observed for generic and CKD specific HRQoL instruments with poorer HRQoL resulting in an increased probability of hospitalisation and mortality [8–10]. Particularly the physical summary score was previously shown to have a strong association with a higher risk of death and may therefore be a subjective, but reliable measure for cardiac dysfunction and cardiac death [8]. Impaired quality of life has not only been recognized as a marker of hypertrophic cardiomyopathy [17] and chronic heart failure [18], but has also been described as cause for developing cardiovascular disorders[19, 20].

In this prospective clinical cohort study we hypothesize that objective functional measures for uremic cardiomyopathy and vasculopathy are associated with subjective measures of quality of life. Patients with essential hypertension and maintained kidney function served as control group.

## Methods

### Study design

Patients aged over 18 years with CKD stage-5 or CKD5d who were either kidney transplant recipients enrolled prior to surgery or transplant waitlisted were included at a tertiary academic clinical centre (University Hospital Coventry and Warwickshire NHS Trust, UK). In parallel a control group was recruited at random from the community through primary care database, comprising healthy individuals being treated for essential hypertension (HTN), without evidence of CKD, diabetes, CVD (heart failure, ischemic heart disease, cerebrovascular disease) or secondary causes for hypertension. In both groups, patients with pre-existing chronic lung disease, inability to cycle due to physical limitation, age over 75 and BMI>38kg/m^2^ were excluded.

Complete study protocol included the SF-36 Health Survey questionnaire, cardiopulmonary exercise testing and clinical assessment (including office brachial blood pressure, echocardiography, vascular tonometry and blood sampling) prior to exercise testing. For patients on hemodialysis, these assessments were carried out on the first non-dialysis day that was at least 12 hours after the last dialysis session in order to avoid the effects of hemodialysis-induced myocardial stunning and minimize the impact of volume load variability on the indices of cardiovascular structure and function [21]. This study was approved by the Black Country Research Ethics Committee (REC:09/H1202/113) and adhered to the declaration of Helsinki. Written informed consent was obtained from all participants.

Between April 2010 and January 2013, we had screened 184 patients with advanced CKD with 143 patients recruited (5 unable to exercise due to physical limitations, 14 did not provide consent and 22 did not complete questionnaire protocol. In the control group, 94 subjects with HTN were screened with 83 recruited (following the exclusion of 5 who had physical comorbid conditions precluding exercise testing, 2 who did not provide consent and 4 did not complete questionnaire protocol). The analysis of HRQoL data presented in this paper was part of a larger study investigating CKD subgroups. (In the larger study the analysis distinguished between pre-kidney transplant, transplant waitlisted CKD patients, and the control group. Recruitment to the three study groups was intended to be 1:1:1, respectively.) However, for the analysis of HRQoL we were only interested in exploring differences between CKD (combined pre-kidney transplant and transplant waitlisted, n = 143) and HTN (n = 83) patients.

### SF-36 short form health survey (version 2)

The SF-36 is the most widely used health related quality of life score worldwide. It is a single item that indicates perceived changes in a person’s health [12]. There are 36 outcomes that can be split into 8 domains: physical function, role physical, bodily pain, general health, vitality, social functioning, emotional role and mental health. Two summary components scores, physical component summarising the first four domains and mental component summarising the latter four domains are also calculated. Domain and summary scores are transformed into a scale 0–100. Higher scores equate to a better health related quality of life. A licence was acquired from QualityMetric Health Outcome Solution, USA (Licence number: QM027853) and calculation software (QualityMetric health outcome^™^ scoring software 4.5) used.

### Cardiopulmonary exercise testing

An experienced blinded investigator performed exercise testing. Cardiopulmonary exercise testing (CPET) was conducted using an electronically braked, upright cycle ergometer to maximal tolerance at an individual work rate. Continuous breath-by-breath analysis (VIASYS, MasterScreen CPX^®^ Hoechberg, Germany) was performed. Equipment was calibrated using standard reference gases and a 3-litre syringe. Each patient rested for 3 minutes followed by 3 minutes of unloaded pedalling prior to workload increments and continuous 12-lead ECG was recorded. All patients continue until symptom limited volitional fatigue. VO_2_peak was measured as the greatest VO_2_ achieved during the final 20-second averaging of peak exercise. Predicted VO_2_peak was determined by the Wasserman and Hansen equation [22]. The V-slope method was used with analyses of ventilatory equivalents and end-tidal gas tension plots in order to calculate VO_2_AT.

### Echocardiography

2-dimensional, Doppler and tissue Doppler transthoracic echocardiography was performed using Vivid 7, GE Healthcare, Horten, Norway ultrasound system according to a standardized study protocol. Calculations included LV ejection fraction according to quantitative biplane Simpson’s method, LV mass and LA volume. Mass and volume measures were indexed to body surface area. Tissue Doppler imaging of the mitral annulus, sequentially at the lateral and septal annular sites were obtained from the apical 4-chamber view. The ratio of early transmitral flow velocity to averaged annular (septal and lateral) mitral velocity (E/mean e') was taken as an estimate of LV filling pressure. All measurements were undertaken according to the American Society of Echocardiography [23] and analysed offline (EchoPac, GE Healthcare).

### Measures of arterial compliance

Pulse wave analysis was performed on the radial artery and carotid-femoral arteries. Pulse wave velocity (PWV) was determined by sequential recording of ECG-gated carotid and femoral waveforms using the high fidelity micromanometer (SPC-301, Miller Instrument, Houston, Texas). An augmentation index adjusted to a heart rate of 75 beats/minute (AIx75) was recorded [24]. Measurements were derived using a validated radial-to-aortic transfer function (SphygmoCor, AtCor Medical Pty Ltd, Australia). All measurements were made in triplicate and mean values used for analysis.

### Statistical methods

Variables are presented as mean, median or frequencies depending on distribution and variable type. Differences in mean values for normally distributed variables were assessed using independent samples t-tests. The Mann Whitney U test was used to assess variables that were not normally distributed. PCS and MCS scores from SF-36 were the outcome variables of interest in this study and thus used as dependant variable in regression modelling analysis. Variables with p-value<0.1 were included in multiple regression analysis. Parameter estimates, standard deviation and 95% confidence intervals were calculated for all regression predictors. An interaction effect estimate is denoted ΔB and can be interpreted as the difference of the slopes of the predictor between groups. All hypothesis tests were two-sided and a p-value of <0.05 was used to indicate statistical significance. SPSS Software Version 22 was used to analyse data.

## Results

### Clinical characteristics

Baseline characteristics of both groups are described in Table 1.

**Table 1:** Characteristics of study populations.

	CKD	HTN	p-value
Total number	143	83	
Male sex, n (%)	89 (62.2)	40 (48.2)	0.08
BMI, kg/m^2^	25.8 ± 4.7	27.5 ± 3.4	0.001
Age, years	46.0 ± 1.1	53.2 ± 0.9	<0.001
Diabetes, n (%)	16 (11.2)	0	-
Hypertension, n (%)	133 (93.0)	83 (100.0)	0.03
*Antihypertensives*			
ACEi/ARB, n (%)	68 (41.0)	51 (58.6)	<0.01
Calcium antagonist, n (%)	87 (52.4)	39 (44.8)	0.3
B-blockers, n (%)	63 (38.0)	12 (13.8)	<0.001
Diuretics, n (%)	27 (16.3)	37 (42.5)	<0.001
CV disease (%)	16 (11.2)	0	-
Smoking, n (%)	73 (51.1)	44 (53)	0.5
Dialysis status, n (%)			
Predialysis	35 (24.5)	-	-
Hemodialysis	91 (63.6)	-	-
Peritoneal dialysis	17 (11.9)	-	-
Biochemistry			
Hemoglobin, g/dl	11.7 ± 1.5	14.2 ± 1.2	<0.001
Urea, mmol/l	18.2 ± 5.9	5.3 ± 1.2	<0.001
Creatinine, μmol/l	655.2 ± 2.0	72.7 ± 14.8	<0.001
eGFR, ml/min/1.73m^2^	9.2 ± 4.5	92.5 ± 15.0	<0.001
Albumin, g/l	43.4 ± 3.8	46.7 ± 2.3	<0.001
Calcium, mmol/l	2.2 ± 0.2	2.2 ± 0.1	0.8
Phosphate, mmol/l	1.6 ± 0.4	1.2 ± 0.3	<0.001
iPTH, pmol/l	36.7 ± 39.5	3.6 ± 1.3	<0.001
hs-CRP, mg/l	7.3 ± 15.8	2.4 ± 2.3	<0.001
NT-pro-BNP, pmol/L	576.9 ± 1345.9	8.3 ± 7.3	<0.001
Troponin-T, μg/L	36.1 ± 33.3	5.1 ± 2.4	<0.001

CKD = chronic kidney disease stage 5 or 5d; HTN = only hypertensive; BMI, body mass index; CV, cardiovascular; eGFR = estimated glomerular filtration rate; iPTH = intact parathyroid hormone; hs-CRP = high sensitive C-reactive protein, NT-pro-BNP = N-terminal prohormone of brain natriuretic peptide. Data are mean values with standard deviation (SD) or total count in sample with percentage (%). P-value by independent-samples t-test (continuous variables) or χ^2^ (categorical variables).

CKD subjects had a lower BMI (25.8 vs. 27.5kg/m^2^, p = 0.001), were younger (46.0 vs. 53.2years, p<0.001), received more B-blockers (38.0 vs 13.8%, p<0.001) for the treatment of hypertension than HTN cohort. In contrast more HTN patients received ACEi/ARB (68.6 vs. 41.0%, <0.01) and diuretics (42.5 vs. 16.3%, p<0.001) than CKD patients. Hemoglobin (11.7 vs. 14.2g/dl, p<0.001) and albumin (43.4 vs. 46.7g/l, p<0.001) were lower but phosphate (1.6 vs. 1.2mmol/l, p<0.001), iPTH (36.7 vs. 3.6pmol/l, p<0.001), hs-CRP (7.3 vs. 2.4mg/l, p<0.001), NT-pro-BNP (576.9 vs. 8.3pmol/L, p<0.001) and Troponin-T (36.1 vs. 5.1μg/L, P<0.001) were higher in the CKD patients compared to the HTN group.

#### SF-36 short form health survey score

Full breakdown of physical and mental component scores are described in Table 2.

**Table 2:** SF 36 score breakdown in the study cohorts.

	CKD	HTN	p-value
**Physical Component Score**	45.0 ± 9.3	53.9 ± 5.5	<0.001
Physical Function	74.6 ± 21.2	94.4 ± 8.7	<0.001
Role Physical	62.4 ± 31.2	91.7 ± 17.0	<0.001
Bodily Pain	70.2 ± 27.0	81.0 ± 23.0	0.002
General Health	45.0 ± 20.8	70.2 ± 13.7	<0.001
**Mental Component Score**	46.9 ± 11.1	54.9 ± 7.6	<0.001
Vitality	44.7 ± 22.6	71.4 ± 17.1	<0.001
Social Functioning	66.8 ± 30.0	92.9 ± 16.5	<0.001
Role Emotional	79.4 ± 26.2	95.0 ± 16.1	<0.001
Mental Health	71.6 ± 20.2	83.1 ± 14.3	<0.001

Data are mean values with standard deviation (SD). Physical component score is a composite score for the subdomains physical function, role physical, bodily pain and general health. Mental component score is a composite score for the subdomains vitality, social functioning, role emotional and mental health.

**Fig 1:**
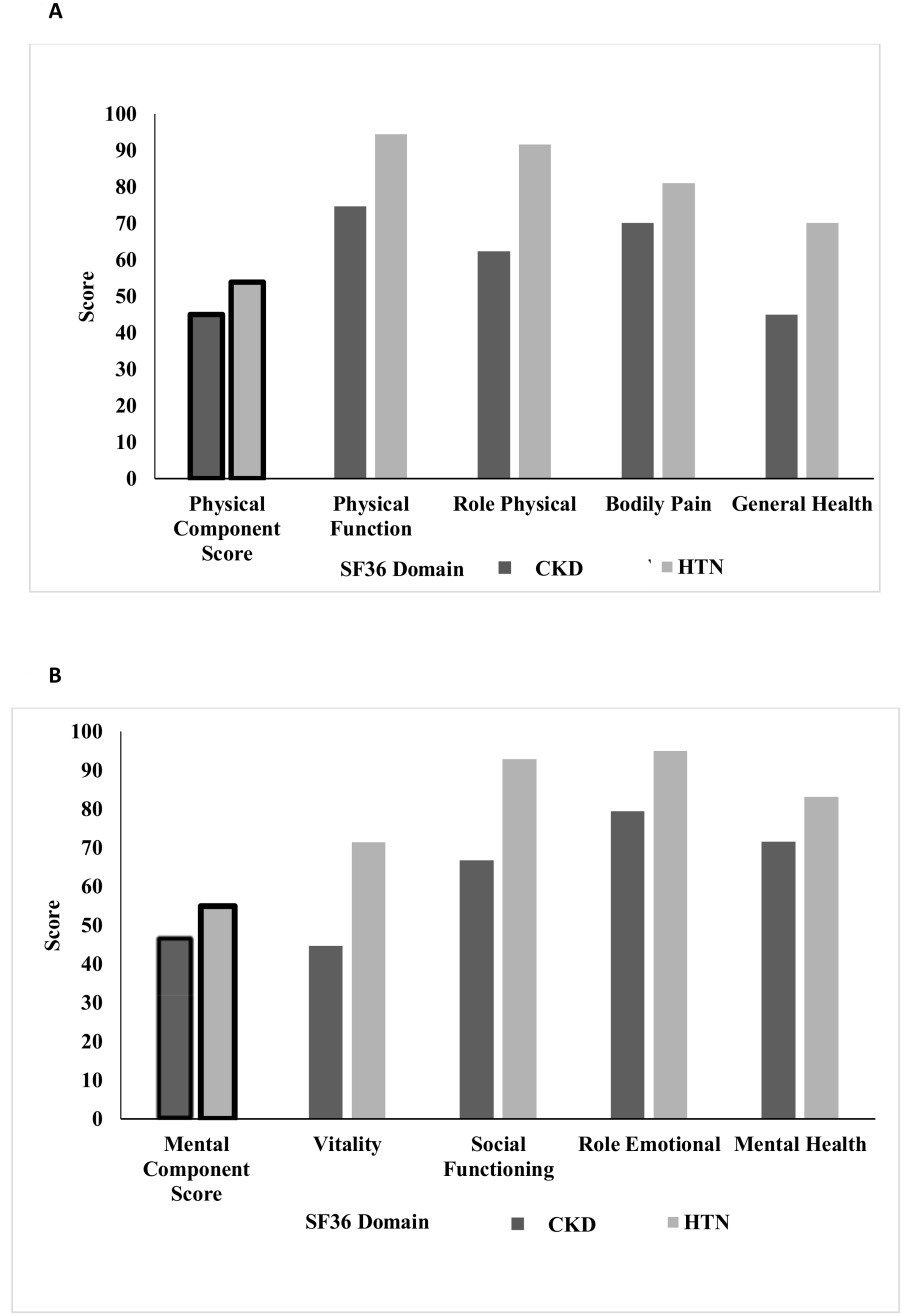
**A**. Physical Component Summary Score and breakdown by domain in CKD and HTN cohorts. **B**. Mental Component Summary Score and breakdown by domain in CKD and HTN cohorts.

The CKD cohort scored significantly lower than HTN patients for PCS (45.0 vs 53.9, p<0.001) and MCS (46.9 vs. 54.9, p<0.001). Patients with CKD reported lower physical function (74.6 vs. 94.4, p<0.001), physical role functioning (62.4 vs. 91.7, p<0.001), increased bodily pain (70.2 vs. 81.0, p = 0.002) and reduced general health (45.0 vs. 70.2, p<0.001) compared to the HTN cohort. A similar pattern was observed for individual domains of the mental score. Graphical representations of these values are shown in Fig 1.

#### Measures of cardiovascular function

Cardiopulmonary exercise, echocardiographic and applanation tonometric findings of the two groups are summarized in Table 3.

**Table 3:** Measures of cardiovascular function in the study cohorts.

	CKD	HTN	p-value
**CPET**			
VO_2_peak, ml/kg/min	19.9 ± 5.4	25.0 ± 7.2	<0.001
VO_2_peak, % of predicted	68.4 ± 16.5	95.5 ± 20.7	<0.001
VO_2_AT, ml/kg/min	11.7 ± 2.3	14.8 ± 3.9	<0.001
VO_2_AT, % of predicted VO_2_peak	40.6 ± 9.2	56.9 ± 12.2	<0.001
RER at VO_2_peak	1.26 ± 0.1	1.22 ± 0.08	0.01
RER at AT	0.89 ± 0.01	0.94 ± 0.07	<0.001
Maximal work load, Watt	109.8 ± 42.4	155.8 ± 61.9	<0.001
Maximal heart rate, bpm	135.5 ± 25.0	155.0 ± 18.7	<0.001
Maximal heart rate, % of predicted	78.1 ± 13.9	93.0 ± 9.9	<0.001
**Echocardiography**			
LV ejection fraction, %	60.7 ± 9.0	66.1 ± 6.0	<0.001
LV mass index, g/m^2^	110.4 ± 36.2	87.8 ± 17.0	<0.001
Left atrial volume index, ml/m^2^	27.7 ± 14.3	25.1 ± 7.6	0.08
E/mean e’	8.9 ± 3.6	8.2 ± 1.8	0.07
LV geometry, n (%)			<0.001
Normal geometry	31 ± 22.4	27 ± 32.5	
Concentric remodelling	59 ± 41.2	41 ± 49.4	
Concentric hypertrophy	55 ± 38.4	7 ± 8.4	
Eccentric hypertrophy	21 ± 14.7	8 ± 9.6	
**Arterial measures**			
Alx75, %	23.1 ± 13.5	26.2 ± 12.1	0.07
PWV, m/s	8.6 ± 2.6	9.0 ± 2.0	0.1
Systolic blood pressure, mm Hg	136.1 ± 20.7	141.2 ± 13.4	0.03
Diastolic blood pressure, mm Hg	80.1 ± 12.0	85.3 ± 9.8	<0.001
Mean arterial pressure, mm Hg	98.8 ± 13.4	104.0 ± 1.0	<0.001

Data are mean values with standard deviation (SD). VO_2_peak, oxygen consumption at maximal exercise / kg body weight; VO_2_AT, oxygen uptake at the point of anaerobic threshold / kg body weight; RER, respiratory exchange ratio; LV, left ventricle; Alx75, augmentation index corrected for heart rate of 75 beats/min; PWV, pulse wave velocity; E/mean e', the ratio of peak early transmitral ventricular filling velocity to the averaged septal and lateral annular mitral velocity.

The CKD group had significantly poorer exercise tolerance and cardiorespiratory performance compared with HTN for maximal oxygen uptake (VO_2_peak: 19.9 vs 25.0ml/kg/min, p<0.001), anaerobic threshold (VO_2_AT: 11.7 vs. 14.8ml/kg/min, p<0.001), maximal work load (109.8 vs 155.8Watt, p<0.001) and maximal heart rate (125.5 vs. 155.0bpm, p<0.001; 78.1 vs 93.0% of predicted, p<0.001).

LV mass index was significantly higher in the CKD than the HTN group (110.4 vs. 87.8g/m^2^, p<0.001). Criteria for LV hypertrophy were present more frequently in CKD patients than the HTN subjects (53.1 vs. 18.1%, p<0.001). The latter group had a higher LV ejection fraction (66.1 vs. 60.7%, p<0.01) than the CKD patients. The measures of vascular compliance were not significantly different in both groups. However, mean arterial pressure (MAP) was lower in the CKD compared to the HTN group (98.8 vs. 104.0mmHg, p<0.001)

#### Independent predictors of the physical component score from the SF-36 health survey

Univariate linear regression analyses for the two groups are presented in Table 4.

**Table 4:** Univariate regression analysis of physical component score in the study cohorts.

Predictor	CKD			HTN		
	*b*	95% CI	p-value	*b*	95% CI	p-value
Smoking	-1.49	-3.55–0.57	0.2	-0.46	-1.89–0.98	0.5
BMI	-0.17	-0.50–0.16	0.3	-0.16	-0.51–0.19	0.4
Diabetes	-2.78	-7.66–2.11	0.3	-	-	-
Hypertension	0.21	-5.85–6.27	0.9	-0.86	-11.84–10.12	0.9
CV disease	-0.26	-7.49–2.28	0.3			
Dialysis vintage	0.00	-0.03–0.03	1.0	-	-	-
VO_2_peak	**0.35**	**0.07–0.64**	**0.02**	**0.27**	**0.11–0.42**	**0.001**
VO_2_AT	0.20	-0.48–0.87	0.6	**0.47**	**0.17–0.76**	**0.002**
HR at peak exercise	0.04	-0.02–0.10	0.2	**0.07**	**0.01–0.13**	**0.03**
Max work load	**0.04**	**0.01–0.08**	**0.03**	**0.03**	**0.01–0.04**	**0.01**
AIx_75_	-0.11	-0.22–0.01	0.07	0.03	-0.07–0.13	0.6
PWV	0.04	-0.55–0.63	0.9	-0.14	-0.75–0.47	0.7
MAP	0.03	-0.08–0.15	0.6	**0.13**	**0.01–0.26**	**0.04**
LV mass index	0.01	-0.04–0.05	0.7	0.06	-0.01–0.13	0.09
LV ejection fraction	0.01	-0.16–0.18	0.9	-0.08	-0.29–0.13	0.5
LA volume index	0.00	-0.11–0.11	1.0	-0.02	-0.18–0.14	0.8
E/mean e'	-0.13	-0.56–0.30	0.6	-0.09	-0.79–0.62	0.8
Hemoglobin	0.20	-0.85–1.25	0.7	-0.20	-1.19–0.79	0.7
Creatinine	0.00	-0.01–0.01	0.5	0.02	-0.06–0.11	0.6
Albumin	**0.40**	**0.01–0.80**	**0.04**	-0.10	-0.63–0.42	0.7
Calcium	2.59	-5.45–10.63	0.5	-3.91	-18.38–10.56	0.6
Phosphate	-1.52	-5.20–2.15	0.4	0.77	-3.75–5.30	0.7
iPTH	-0.01	-0.05–0.03	0.5	-0.38	-1.27–0.51	0.4
hs-CRP	-0.01	-0.11–0.10	0.9	-0.21	-0.73–0.31	0.4
NT-proBNP	0.00	-0.01–0.01	0.6	0.05	-0.11–0.22	0.5
Troponin-T	-0.01	-0.06–0.04	0.8	-0.03	-0.54–0.47	0.9

*b*, unstandardized regression coefficient: change in PCS per one unit change of variable.

In the CKD group a positive association was noted between VO_2_peak (*b* = 0.35, p = 0.02), maximum work load (*b* = 0.04, p = 0.03), serum albumin (*b* = 0.40, p = 0.04) and PCS. There was also positive correlation between the PCS and VO_2_peak (*b* = 0.27, p = 0.001), VO_2_AT (*b* = 0.47, p = 0.002), heart rate at maximal exercise (*b* = 0.07, p = 0.03), maximum work load (*b* = 0.03, p = 0.01) and MAP (*b* = 0.13, p = 0.04) in the HTN group. No associations were noted between PCS and echocardiographic measures, vascular elasticity, hemoglobin, biochemical markers of mineral metabolism, inflammation or cardiac markers in either group.

The MCS did not show any relationship with the tested variables in the CKD or HTN group (data not shown).

Table 5 presents multiple regression models adjusted for demographic variables age, sex and BMI, and additionally dialysis vintage in the CKD group.

**Table 5:** Multiple regression analysis of physical component score in CKD and HTN cohorts.

^a^Models	*b*	Standard Error	95% CI	p–value
^†^**Model I: CKD**				
Intercept	36.92	8.76	19.60–54.25	<0.001
Age	0.05	0.06	-0.08–0.17	0.5
Sex	-0.69	1.68	-4.02–2.64	0.7
BMI	-0.04	0.19	-0.42–0.34	0.8
Dialysis vintage	0.00	0.01	-0.02–0.03	0.8
VO_2_peak	0.39	0.18	0.03–0.74	0.03
^†^**Model II: HTN**				
Intercept	57.82	9.55	38.82–76.82	<0.001
Age	-0.18	0.08	-0.33 - -0.03	0.02
Sex	1.63	1.36	-1.07–4.33	0.2
BMI	-0.10	0.17	-0.44–0.25	0.6
VO_2_peak	0.24	0.10	0.04–0.44	0.02

^a^All models of CKD cohort adjusted for demographic variables age, sex, BMI and dialysis vintage; All models of HTN cohort adjusted for demographic variables age, sex and BMI.

^†^Final model derived through variable selection process following initial inclusions of univariates with p<0.1. *b*, unstandardized regression coefficient: change in PCS per one unit change of variable.

VO_2_peak, oxygen consumption at maximal exercise / kg body weight

The unstandardized regression coefficient represents the change in PCS while holding the other variables constant. In the CKD group only VO_2_peak (*b* = 0.39, p = 0.03) was significantly associated with PCS. For the HTN group a similar positive measured effect was observed (VO_2_peak; *b* = 0.24, p = 0.02). Increasing age showed a decline in measured physical performance (PCS) (*b* = -0.18, p = 0.02).

#### The effect of uremia on the physical component score from the SF-36 health survey

**Fig 2:**
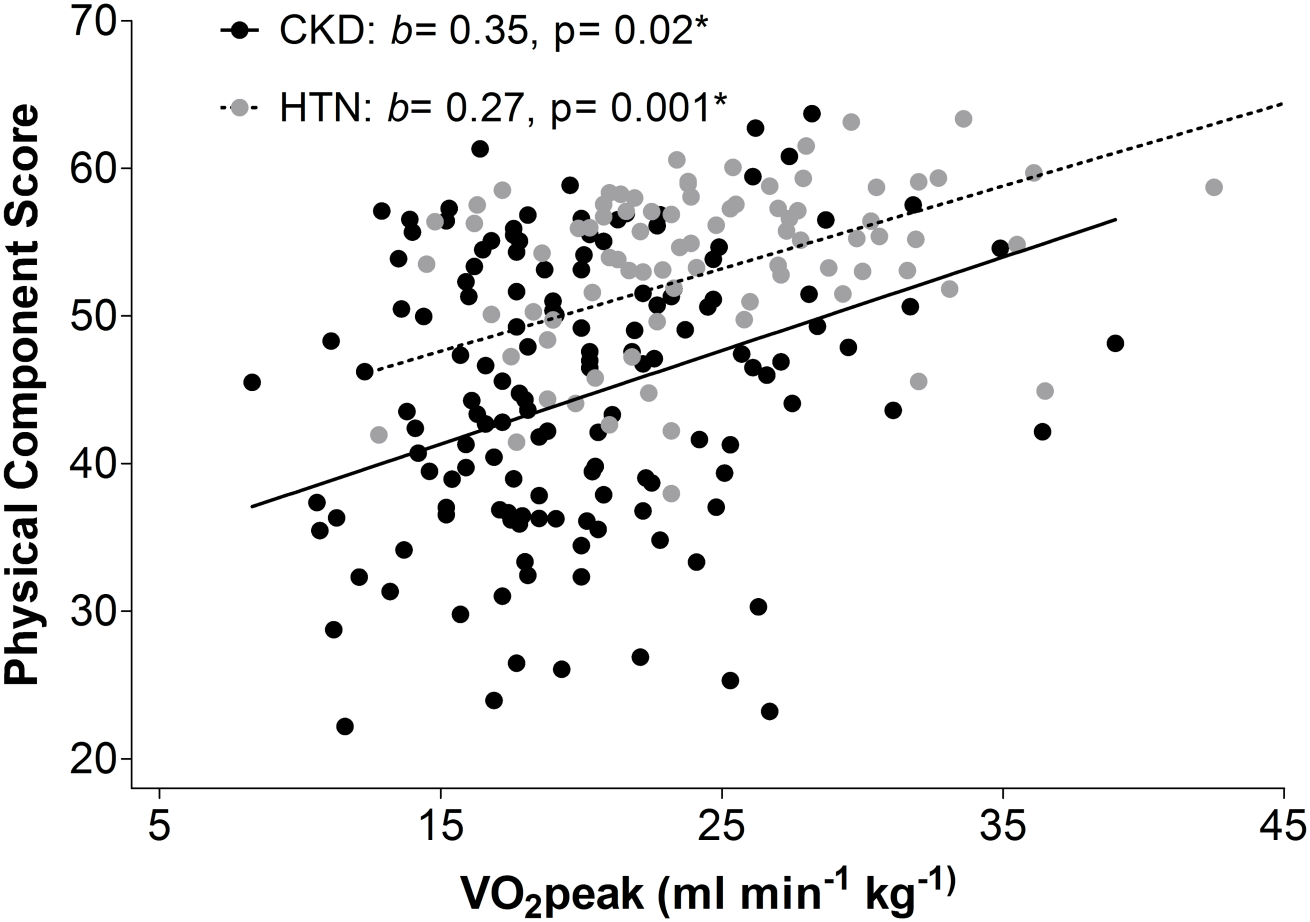
**A** depicts the unadjusted regression of the PCS on VO_2_peak in the CKD and HTN cohorts. Unadjusted regression of Physical Component Score on VO_2_peak in the CKD and HTN cohort. *b*, unstandardized regression coefficient: change in PCS per one unit change of variable. *p–value<0.05. Dash line = HTN, straight line = CKD. **B** demonstrates the same regression after adjustment for age, sex and BMI. Lack of difference of changes in VO_2_peak with Physical Component Score between the CKD and HTN cohorts. *ΔB* is the difference in the parameter estimates between the regression lines for the HTN and CKD groups. Group interaction with VO_2_peak was adjusted for age, sex, and BMI. Dash line = HTN, straight line = CKD.

There is no significant difference in the change of PCS with VO_2_peak between the 2 groups (ΔB = 0.08; p = 0.7). However, lower PCS for a given VO_2_peak value in the CKD group compared to the HTN group (Fig 2) suggest that the effect of uremia on cardiopulmonary function characterised through a reduced VO_2_peak is perceived in a quantifiable reduction of physical ability.

## Discussion

The interaction between HRQoL measures and pathophysiological processes are complex. Our prospective cohort study showed a clear positive relationship between objective integrated functioning for the pulmonary, cardiac, circulatory and muscle metabolic system measured with CPET and self-reported measures of HRQoL in all study patients. We demonstrated that VO_2_peak is a strong predictor of PCS for both CKD and HTN cohorts. Although the correlations between VO_2_peak and PCS were similar between the CKD and HTN patient cohort, at a given VO_2_ peak the perceived physical score was significantly lower in the former group. To our knowledge this is the first description of a clear link between a standardised objective measure of global biological function and HRQoL.

We found HRQoL measures to be significantly reduced for both PCS and MCS in our CKD cohort, confirming previous observations [8–10] from similar CKD patient cohorts. Our scores were somewhat higher than other dialysis cohorts[8, 10] probably due to our inclusion of CKD patients fit enough to undergo a renal transplant. Hypertension is prevalent in CKD and modulates cardiovascular function [14]. Therefore we selected patients with treated essential hypertension as a control group to the CKD population to investigate the potential role of uremia itself on the interaction between subjective and objective functional measures in CKD. Essential hypertension itself reduces quality of life measures [25] and impairs exercise tolerance [26] when compared to healthy individuals. Significantly reduced quality of life measures in CKD compared to HTN patients were reflected in much lower measures of cardiovascular function in CKD patients as previously described[14, 27, 28].

Apart from serving indirectly as a marker for other prognostic factors, VO_2_peak directly reflects the capacity to increase cardiac output, pulmonary function and skeletal muscle oxygen utilisation in response to the physiologic stress of exercise. It is therefore plausible to expect a correlation of VO_2_peak with patient experience of physical function in daily life as demonstrated in our study. Reduced exercise tolerance is in part due to potentially reversible, functional causes like physical deconditioning. It is encouraging that exercise programs are able to improve exercise tolerance and in parallel quality of life, in particular physical functioning in patients with advanced CKD[15, 29, 30],[31, 32] and patients with essential hypertension [33] supporting a direct link between biological objective and perceived subjective measurements. In fact physical inactivity likely contributes to increased morbidity and premature mortality for patients with CKD[33, 34].

The capacity to transport oxygen is determined by cardiovascular function and hemoglobin concentration. Reduced cardiovascular capacity and hemoglobin concentration have been shown to determine morbidity, mortality and HRQoL for patients with CKD[13, 14, 35–37]. For HTN patients HRQoL was improved when blood pressure was controlled [25]. We did not find a correlation for echocardiographic or molecular measures of cardiac function, vascular elastance or hemoglobin concentration in the CKD or HTN cohort. Measures for cardiovascular function in a cross-sectional not preselected CKD population are usually found to be lower[14, 38] compared to our CKD patients. However, LV ejection fraction does not correlate with HRQoL measures even in patients with congestive heart failure [39]. High Troponin-T but not NT-pro-BNP serum levels are associated with lower scores in physical function and vitality domains from SF-36 in advanced CKD patients without symptomatic cardiac disease or LV hypertrophy [40]. When using multiple regression analysis VO_2_peak remained a predictive measure for PCS in both patient groups. CPET as the gold standard global exercise test is likely measuring most aspects of physical HRQoL experience more accurately and is more sensitive, a notion supported by the observation that all CPET measures correlate with PCS for the HTN cohort.

The capacity for utilization of oxygen is related to skeletal muscle mass and function, which are dependent on nutritional status. A measure for nutritional status is serum albumin. It predicts morbidity and mortality, but also quality of life, particularly of patients with CKD[9, 41]. Here we observed an association of albumin and PCS in CKD. On the contrary, we did not observe a significant association between BMI and PCS among the CKD subjects despite the recognition of higher BMI being paradoxically advantageous in advanced CKD[42, 43]. This could be explained by the significantly lower BMI measured among the CKD subjects compared to the HTN controls, suggesting the common policy of precluding patients from transplant listing based on the BMI criterion. Major clinical risk factors such as diabetes, hypertension or smoking also did not significantly predict the PCS but this could simply reflect the limitation of using single surrogate marker as independent risk predictor[13, 44].

In both study cohorts, VO_2_peak was an independent predictor of PCS. However, it is interesting to note that after adjusting the regression of PCS on VO_2_peak for demographic characteristics gender, age and BMI there was no difference in the parameter estimates between the regression lines. This suggests that PCS is a reliable composite predictor of CPET measures and that the pathophysiological mechanism, treated hypertension with or without uremia did not alter the correlation between functional measures and subjective experience. It is also intriguing to note that for a given VO_2_peak the experienced PCS was much lower for patients with CKD than for patients with HTN. Likely reasons are the systemic processes and effects caused by CKD, uremia and also treatment modality resulted in reduced exercise tolerance with associated reduced HRQoL. Blunted chronotropic and inotropic response to exercise [14], cardiac stunning [21], decreased oxygen delivery [45], pulmonary congestion [46], anemia [47], and skeletal muscle atrophy modulated by the upregulation of the IGF-1 signalling pathway [48] have been described as potential mechanisms affecting CKD patients beyond hypertension. Daily hemodialysis somewhat improves the perceived uremic effects [49] but more comprehensive reversal of CKD effects are experienced by patients receiving a renal transplant [13].

Although the difference in VO_2_peak score between the two cohorts can explain a large part of the discrepancy in HRQoL measured, other bio-psychosocial factors could also contribute to the impaired HRQoL. Reduced exercise tolerance, other symptoms of uremia and frequent hospital visits for dialysis can all contribute[1, 7]. As CKD progresses through from stage 1 to 5, all HRQoL dimensions have been shown to deteriorate. The largest deficits occur in PCS and the smallest in mental health and bodily pain. Patients with CKD, on dialysis, have the lowest scores across the stages in every domain [50]. Hemodialysis patients have lower QoL scores compared with the general population in all dimensions[1, 50] but renal transplant appears to reverse this deficit to scores near to general population norms[51].

In both CKD and HTN groups, the demographic, clinical and molecular variables tested had no significant effect on the MCS[1, 5, 50]. This is in line with the literature in that clinical variables have minimal effect on MCS in CKD[5, 50]. It is worth noting that MCS scores were significantly lower in CKD than HTN. Mental well-being in patients with CKD can be affected by multiple stressors. Fatigue, sleep disturbances, anorexia and cognitive dysfunction are associated symptoms of uremia that can contribute to this [1]. For every ten point reduction in MCS, there is an associated increase in mortality by 28%^7^.

## Limitations

HRQoL is a subjective, very complex and dynamic process that is difficult to capture completely in a single measure. Although we used the established HRQoL SF-36 questionnaire, inclusion of CKD specific questions could have provided additional depth. Larger patient cohorts are needed to break into each domain. Our findings are limited to patients with advanced CKD and therefore further studies would need to assess if these findings can be supported across all stages of CKD.

## Conclusion

For patients with CKD or HTN objective physical performance had a significant effect on self-reported physical health and functioning. Self-reported physical health and functioning is a reliable and useful tool to direct exercise programmes, transplant work-up and screen for risk.

## Supporting information

S1 Dataset Detailed listing of results cited in this paper. Data Repository: BioStudies Ascension number S-BBST34.
(XLSX)
