# Correction: Quality of life measures predict cardiovascular health and physical performance in chronic renal failure patients

**DOI:** 10.1371/journal.pone.0189382

**Published:** 2017-12-05

**Authors:** A. Rogan, K. McCarthy, G. McGregor, T. Hamborg, G. Evans, S. Hewins, N. Aldridge, S. Fletcher, N. Krishnan, R. Higgins, D. Zehnder, S. M. Ting

Panel B is missing from Fig 2. Please view the complete Fig 2 here.

**Fig 2:**
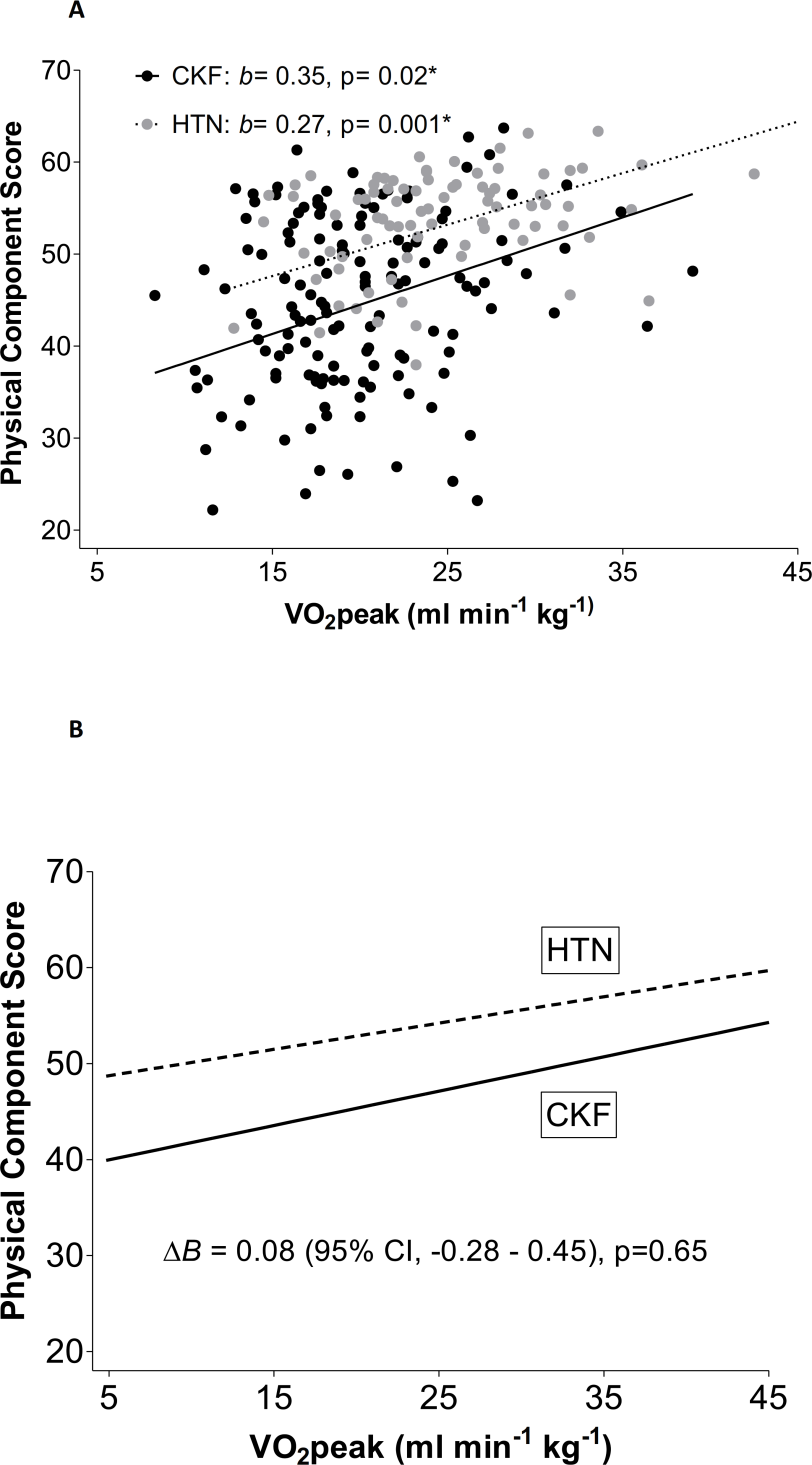
**A** depicts the unadjusted regression of the PCS on VO_2_peak in the CKD and HTN cohorts. Unadjusted regression of Physical Component Score on VO_2_peak in the CKD and HTN cohort. *b*, unstandardized regression coefficient: change in PCS per one unit change of variable. *p–value<0.05. Dash line = HTN, straight line = CKD. **B** demonstrates the same regression after adjustment for age, sex and BMI. Lack of difference of changes in VO_2_peak with Physical Component Score between the CKD and HTN cohorts. *Δ*B is the difference in the parameter estimates between the regression lines for the HTN and CKD groups. Group interaction with VO_2_peak was adjusted for age, sex, and BMI. Dash line = HTN, straight line = CKD.
